# Stronger Syntactic Alignment in the Presence of an Interlocutor

**DOI:** 10.3389/fpsyg.2019.00685

**Published:** 2019-03-27

**Authors:** Lotte Schoot, Peter Hagoort, Katrien Segaert

**Affiliations:** ^1^Max Planck Institute for Psycholinguistics, Nijmegen, Netherlands; ^2^Donders Institute for Brain, Cognition and Behaviour, Nijmegen, Netherlands; ^3^School of Psychology, University of Birmingham, Birmingham, United Kingdom; ^4^Centre for Human Brain Health, University of Birmingham, Birmingham, United Kingdom

**Keywords:** syntactic choice, structural priming, alignment, interlocutor, conversation, passives

## Abstract

Speakers are influenced by the linguistic context: hearing one syntactic alternative leads to an increased chance that the speaker will repeat this structure in the subsequent utterance (i.e., syntactic priming, or structural persistence). Top-down influences, such as whether a conversation partner (or, interlocutor) is present, may modulate the degree to which syntactic priming occurs. In the current study, we indeed show that the magnitude of syntactic alignment increases when speakers are interacting with an interlocutor as opposed to doing the experiment alone. The structural persistence effect for passive sentences is stronger in the presence of an interlocutor than when no interlocutor is present (i.e., when the participant is primed by a recording). We did not find evidence, however, that a speaker’s syntactic priming magnitude is influenced by the degree of their conversation partner’s priming magnitude. Together, these results support a mediated account of syntactic priming, in which syntactic choices are not only affected by preceding linguistic input, but also by top-down influences, such as the speakers’ communicative intent.

## Introduction

Conversation partners influence each other’s linguistic choices. What you hear as a listener in one conversation turn influences what you say as speaker in the next (and vice versa). In this paper, we focus on syntactic processing and sentence structure choices. We compare syntactic choice priming effects in conditions with versus without a conversation partner (or, interlocutor) present, to investigate whether only the linguistic context, or also top-down influences - which come into play in the presence of an interlocutor - can affect syntactic choices.

Priming effects in syntactic choices were first reported as a tendency for speakers to repeat their own syntactic choices (production-to-production priming effects or syntactic persistence, Bock, 1986). Since then, a large body of evidence showed that syntactic choices are affected also by structures the speaker heard before (comprehension-to-production priming effects or syntactic alignment: Branigan et al., 2000; Bock et al., 2007). Explanations of the cognitive mechanisms influencing syntactic priming effects have been provided by accounts that focus on implicit learning mechanisms (Chang et al., 2000, 2006; Jaeger and Snider, 2013), residual activation (Pickering and Branigan, 1998) or a combination of these (Reitter et al., 2011). Despite differences, these influential accounts share a focus on explaining how *linguistic* context influences syntactic choice.

However, others have proposed that when syntactic priming effects are studied in a conversation, there may be additional, *top-down* factors that influence how much speakers align with their partner, such as the speakers’ social and communicative goals (Giles and Powesland, 1975; Balcetis and Dale, 2005; Branigan et al., 2010; Coyle and Kaschak, 2012; Weatherholtz et al., 2014; Schoot et al., 2016; for a review: see Segaert, 2019). The latter findings suggest a mediated account of syntactic alignment (Branigan et al., 2010), where the degree of priming magnitude can be modulated by top-down influences. The aim of the present study is to add empirical evidence on whether top-down influences affect the degree to which syntactic priming occurs, with the aim to ultimately further shape accounts of syntactic priming and language production.

The first hypothesis we test in this study, is whether the degree of syntactic alignment is influenced by being in a conversational context. Crucially, being in a conversational context implies the presence of a conversation partner, or, interlocutor. We investigate syntactic alignment in the presence versus absence of an interlocutor. When an interlocutor is present, a speaker has the intention to communicate a message to an addressee. If it is the case that top-down influences such has having a communicative intent, can shape cognitive processing of the speaker, then the degree of syntactic alignment might be affected. On the other hand if syntactic alignment is purely a low level, automatic effect of priming the sentence structure, then the strength of syntactic alignment should be the same whether or not the speaker has a communicative intent.

There is some reason to believe that the presence/absence of communicative intent could influence the magnitude of syntactic priming effects. To facilitate communication, speakers often adapt what they say or how they say it (audience design: Bell, 1984). Syntactic alignment may be one way of facilitating comprehension for a conversation partner. By aligning with their partner’s sentence structures, speakers are likely to facilitate comprehension for the listeners. Indeed, several studies indicate that language comprehension is facilitated when syntax is repeated (Noppeney and Price, 2004; Branigan et al., 2005; Arai et al., 2007; Thothathiri and Snedeker, 2008; Weber and Indefrey, 2009; Menenti et al., 2011; Ferreira et al., 2012; Segaert et al., 2012; Schoot et al., 2014). Intuitively, then, we may hypothesize that when an interlocutor is present, speakers may (unconsciously) try to facilitate their partner’s comprehension process by repeating their syntactic choices back to them.

Some studies provided indirect evidence in support of this hypothesis. For example, in the first study on syntactic alignment in dialogue, Branigan et al. (2000) state that syntactic priming effects in dialogue are much larger than effects found in monologue studies, although this was not empirically tested. Reitter et al. (2006) suggest that the more important it is that communication is smooth and efficient, the more speakers seem to align their syntactic structures with their partner. This could perhaps be explained by a desire to facilitate comprehension for the listener. In a different line of studies, Branigan et al. (2003) found that speakers align their syntactic (and lexical, see Branigan et al., 2011) choices more in a situation where the interlocutor benefits more from audience-targeted, adapted language use because they are less likely to understand what the participant is saying (Branigan et al., 2010). These studies suggest that speakers align their linguistic choices with their partner at least in part with the aim to facilitate comprehension for that partner.

In a conversation context with an interlocutor present, social aspects of the interaction may also come into play. These are interpersonal feelings and opinions that the speaker has of the conversation partner, or a desire to be liked by the conversation partner. All of these may modulate the degree of alignment between interlocutors (for a review: Segaert, 2019) but were not explicitly manipulated in the present study. We focused our interests on the top-down influence of having an interlocutor present. We compare this to a context where participants receive the same linguistic input but no interlocutor is present. Any differences found between conditions in which an interlocutor is present vs. absent, can at least to some degree be attributed to the presence vs. absence of communicative intent.

In addition to our main manipulation, we also manipulate the alignment behavior of the “partner” (whether this partner is an interlocutor or merely pre-recorded descriptions). The alignment behavior of a conversation partner may influence the degree of syntactic alignment observed for a speaker. In our study, participants either interact with a partner who consistently aligns or repeats the participants’ syntactic choices, or a partner who does not align or repeat their syntactic choices. This manipulation is explorative, mainly because previous work on syntactic alignment has approached the effect from a somewhat individualistic perspective. Most often, the other speaker in a syntactic priming experiment is a scripted confederate who provides primes *for* the participant, but cannot be primed *by* the participant. In natural conversation, however, there are two naïve “participants.” This means that speakers would not only be primed by their partner, this partner would also be primed by them. We test the exploratory hypothesis that speakers align more with partners who repeat their structural choices. Such an effect could be driven by multiple mechanisms: speakers may like partners who align their syntactic structures more than partners who do not align with them (see for example Van Baaren et al., 2003 and Abrahams et al., 2018 on the influence of language mimicry on prosocial behavior), which may in turn influence the speakers’ own syntactic alignment. Alternatively, it might be a reciprocal effect (see Schoot et al., 2014): if you facilitate communication for me, then I will do my best to facilitate comprehension for you. The goal of the current study, however, is merely to establish whether the alignment behavior of the partner indeed affects the degree to which speakers align with their partner. We do not aim to dissociate between different mechanistic explanations, but rather to provide an initial step toward a more naturalistic syntactic priming paradigm.

In the experiments described below, we measure the effect of syntactic priming on participants’ syntactic choices (actives vs. passives). We predict that participants will produce more passive targets following a passive comprehension prime than following a baseline prime (i.e., inverse preference effect in syntactic choice priming, Ferreira and Bock, 2006). We additionally test the following two hypotheses. First, we test whether the degree of syntactic alignment is influenced by the presence versus absence of an interlocutor. To that end, we compare syntactic alignment for participants who interact with a physically present interlocutor (i.e., a confederate), to participants who describe photographs and listen to pre-recorded descriptions (Interlocutor vs. No Interlocutor Condition). We hypothesize that speakers align more in the presence of an interlocutor. Orthogonal to the first manipulation, we also manipulated how much the “partner” (i.e., either the interlocutor or pre-recorded descriptions) aligned their syntactic choices with the participant (Adaptive vs. Non-Adaptive Condition), to test our second hypothesis that speakers align more with partners who repeat their structural choices.

## Materials and Methods

### Participants

All participants were Dutch native speakers who were not color-blind and had no language or speech disorders. They were compensated financially for their participation and gave written informed consent in accordance with the declaration of Helsinki. The study was approved by the local Ethics Committee of the Social Sciences faculty of the Radboud University (Ethics Approval Number ECG2013-1308-120).

#### Interlocutor Conditions

Sixty-nine participants were assigned to the interlocutor conditions (either Adaptive or Non-adaptive). Nine participants were excluded from the analyses. One of them did not believe the interlocutor (i.e., a confederate) was a naïve participant and another described all photographs with the same strategy, naming the left actor first. The remaining seven participants did not produce any passive descriptions following intransitive primes, which prevented us from manipulating the confederate’s priming magnitude (thus creating an Adaptive versus Non-Adaptive condition). Half of the 60 included participants were assigned to the Adaptive Interlocutor condition (*N* = 30, 10 male, M_age_: 21.1 years, SD_age_: 2.96) and half to the Non-Adaptive Interlocutor condition (*N* = 30, 10 male, M_age_: 20.9 years, SD_age_: 2.55).

#### No Interlocutor Conditions

Sixty participants participated in these conditions, but four were excluded from the analysis: one participant did not complete the experiment due to illness; two did not produce any passive descriptions following intransitive primes; the last was excluded because in all priming conditions, passive target production was more than 3 SD above the group mean. Twenty-nine participants were assigned to the Adaptive No Interlocutor Condition (8 Male, M_age_: 22.4 years, SD_age_: 2.74), and 27 participants to the Non-adaptive No Interlocutor condition (5 male, M_age_: 21.06 years, SD_age_: 2.26).

The results reported in this paper have already appeared in the Ph.D. thesis of the LS (Schoot, 2017), which can be accessed online at http://hdl.handle.net/2066/166360.

### Task and Design

**Figure 1:**
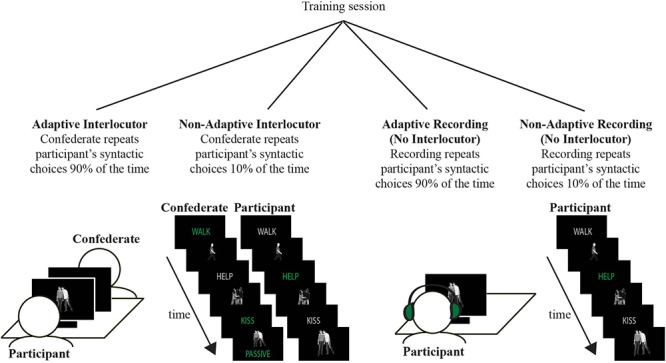
Study design: participants conducted a syntactic priming experiment in one of 4 conditions: Adaptive Interlocutor, Non-Adaptive Interlocutor, Adaptive No Interlocutor, Non-Adaptive No Interlocutor. The experiment set-up for the Interlocutor versus No Interlocutor conditions are illustrated on the left versus right, respectively. The experiment was in Dutch, we use English translations in the figure to help the reader.

In all four conditions (the manipulations are explained below), participants played a simple picture description game, consisting of alternating comprehension and production trials (illustrated in Figure 1). During production trials, participants were instructed describe the photograph, using a concise sentence containing the verb that was presented immediately preceding the photograph. During comprehension trials, participants listened to a description of the photograph and decided whether the photograph on their screen matched the description they heard. Participants used a button press for comprehension trials if there was a mismatch between photograph and description (occurring on 20% of the filler trials).

There were two orthogonal between-participant manipulations: Interlocutor vs. No Interlocutor and Adaptive versus Non-Adaptive partner (explained below). This resulted in four versions of our experiment, within which we measured the effect of syntactic priming on transitive sentence production.

### Interlocutor vs. No Interlocutor Conditions

All participants assigned to the Interlocutor conditions interacted with the same female confederate, whom they believed to be another naive participant (as verified during a debrief). The confederate and the participant were sitting opposite each other, both facing a computer screen (see Figure 1), and took turns describing photographs. On the other hand, in the No Interlocutor conditions, participants did not talk to anyone during production trials and during comprehension trials the photographs were accompanied by pre-recorded descriptions.

To ensure the same degree of experimental control in the Interlocutor and No Interlocutor conditions, the confederate was not free in how she described the pictures. On the confederate’s computer screen, transitive photographs were always accompanied with the word “active” or “passive” (pre-programmed). The confederate was instructed to describe the photograph with an active or a passive sentence using the verb presented immediately preceding the photograph. Crucially the participant was led to believe that the confederate was also freely describing the photographs. The confederate was well trained and made no mistakes, which was verified by the experimenter who was present.

As an additional measure to avoid suspicion about the naivety of the confederate, both participant and confederate were instructed to detect mismatches. Participants were instructed to press the left mouse button when they detected a mismatch, after which they heard a beep. The beep also played when the interlocutor (confederate) detected a mismatch. We created mismatches by presenting different photographs to confederate and participant. Half of the mismatches had to be detected by the participant and half by the confederate. In the No Interlocutor conditions, all mismatches were detected by the participant.

To further increase the contrast between the Interlocutor and No Interlocutor conditions, participants got feedback concerning their performance on the mismatch detection task. In the No Interlocutor conditions, the score was based merely on the participant’s individual performance during comprehension trials. In the confederate conditions, however, the experimenter would stress that both “participants” (participant and confederate) should work together to increase their score. The performance score reflected a team effort: pairs could only achieve a good performance if they described the pictures correctly to their partner *and* paid attention to what their partner was saying.

### Adaptive vs. Non-adaptive Conditions

Fifty manipulation trials were included in the experiment to enable us to have an Adaptive versus Non-Adaptive partner. The sentence structure used to describe these trials was manipulated online. In the Adaptive conditions, the participant’s marked syntactic choice (i.e., passive sentence production following a baseline prime) would consistently (in 90% of the cases) be repeated back to them in the next trial (i.e., the manipulation trial). In the Non-Adaptive conditions, the participant’s marked syntactic choice would rarely be repeated in the following manipulation trial (only in 10% of the cases). For active targets produced by the participant, there was no difference between the two conditions: actives were repeated for 90% of the cases.

Importantly, we ensured that there was no between-group difference in the total number of passives that participants heard between Adaptive and Non-Adaptive conditions. We made sure that on average 7.5 additional transitive fillers were described with a passive in the Non-Adaptive conditions (7,5 was the average number of passive manipulation trials in the Adaptive conditions).

#### Pre-experiment Training Session

Since the Adaptive/Non-adaptive manipulation hinges on participants producing passive target descriptions following intransitive primes, we added a training session to the experimental procedure. Previous studies have shown that such a training session increases the chance that participants produce passive targets in the main experiment (Kaschak et al., 2006; Segaert et al., 2011).

The training session was kept as similar as possible in all four conditions: participants always did the training together with a physically present partner. In the Interlocutor condition, this partner was the confederate. In the No Interlocutor condition, participants did the training session together with another participant, after which they would both proceed to participate in the main experiment individually.

In the training session, participants were presented with 120 photographs in alternating comprehension and production trials. The production photographs were color-coded (note that this is different from the main experiment production trials): one of the figures was colored red, the other green. Participants were instructed to always name the green figure before the red figure (stop light paradigm, Menenti et al., 2011; Segaert et al., 2011). For 90% of the transitive photographs, the patient was colored green and the agent red, resulting in a passive sentence (e.g., “The woman is hugged by the man”). For the other 10% of the trials, the agent was green and the patient was red, resulting in an active sentence (e.g., “The man hugs the woman”). Each participant saw a unique list of photographs and no participant saw one photograph more than once. Presentation order was randomized.

### Trial Types and List Composition of Main Experiment

All participants were presented with 210 comprehension trials and 210 production trials. The first trial for every participant was a comprehension trial, after which comprehension and production trials alternated. There were four types of trials:

– Production target trials: participants described 100 transitive photographs. For these, the participant was free to describe the photograph with a sentence in the active or in the passive voice.
– Comprehension prime trials: These preceded production target trials. There were 50 transitive primes, (25 were *active primes* and 25 were *passive primes*), and 50 *baseline primes* (these were descriptions of intransitive events).
– Comprehension manipulation trials: Production targets that followed a baseline prime (i.e., 50 targets) were in turn followed by another transitive item (*manipulation trial*). For more information, see Adaptive vs. No Adaptive Condition above.
– Filler trials: Each participant saw 170 filler photographs (115 intransitive, e.g., *the man runs*; 35 locative, e.g., *the ball is on the table;* 20 transitive).

The order in which trials were presented was randomized for each participant, with two main restrictions. First, production targets were always preceded by a comprehension prime. Second, baseline prime – production target pairs were always followed by a manipulation trial. Furthermore, for each prime structure (active or passive), half of the items were presented in the first part of the experiment and the other half in the second part of the experiment (separated by a break). For each participant, photographs were randomly chosen from the database with the restriction that individual photographs could not appear more than once in each list. Actions could be repeated within a list, but only when depicted by different actors or with the same actors assigned to different thematic roles. Every 40 trials, participants were presented with a feedback screen with the percentage of trials to which they had responded correctly.

### Materials

The photographs have been described extensively elsewhere (e.g., Segaert et al., 2011) but briefly: there were transitive, intransitive and locative photographs. Transitive photographs depicted two actors performing a transitive action (e.g., kissing, serving). Actor pairs either consisted of two adults or two children, and there was always one male and one female actor in the photograph. There were photographs of two pairs of children and two pairs of adults for each depicted action, each once with the female as agent and once with the male as agent. Intransitive photographs depicted one actor performing an intransitive action (e.g., walking). Locative photographs depicted two objects and could be described with a locative state sentence (e.g., “the keys lie on the table”) or a frontal locative (e.g., “on the table lie the keys”). For each photograph, descriptions were recorded by a female Dutch native speaker (all descriptions were in Dutch). For transitive photographs, there was one recording of a description in the active voice and one in the passive voice. These recordings were presented in the No Interlocutor condition only, since in the Interlocutor condition, they were described by the confederate.

### Trial Structure

Each trial (comprehension or production) started with a blank screen (duration of which was jittered between 0 and 1000 ms), after which the verb was presented for 500 ms. The color of this verb indicated whether a production (verb is green) or a comprehension (verb is gray) photograph was coming up. After an interval jittered between 500 and 2500 ms (in which a blank screen was presented), participants were presented with a photograph (on screen for a direction of 2000 ms). For comprehension trials in the No Interlocutor context, a recorded description of the photograph was played to the participant. The recording started after the picture appeared on the screen, the delay was jittered between 0 and 1000 ms. A blank screen was then presented for a duration jittered between 1000–4000 ms, before the next trial started (7 s total trial time).

### Procedure

In the Interlocutor conditions, participant and confederate were picked up from the waiting room together as to avoid any suspicion about the naivety of the confederate; they then completed the training session and main experiment together. After the first half of the main experiment, there was a break during which participant(s) and confederate got something to eat and drink and interaction was encouraged. After completion of the main experiment, the experimenter checked whether the participant believed the other participant/confederate was also a naive participant. If not, this participant would be excluded.

In the No Interlocutor conditions, participants were invited in pairs and picked up from the waiting room together; they completed the training session in the same room but the main experiment in separate experiment rooms.

During the experiment, the experimenter was not visible to the participants. She coded the utterances online for correctness. An utterance was incorrect if participants did not use the presented verb in their description or when agent and/or patient were not named correctly (e.g., participants said “woman” when a girl was shown). We excluded 0.9% (106 out of 11599) of target responses because they were not described correctly.

The training session took about 11 min; the main experiment took about 50 min. The total session (including reading the instructions and the break) took about 1 h and 45 min.

### Data Analysis Approach

Participants’ syntactic choices were analyzed with a generalized linear mixed effect model, using the glmer function of the lme4 package (Bates et al., 2012) in R (R Core Team, 2013). Target responses were coded as 0 for actives and 1 for passives. Incorrect responses (actors or action not named correctly) were not analyzed. Our model included fixed effects for the categorical predictor variables *Prime Structure* (active / passive / intransitive), *Partner* (interlocutor / no interlocutor) and *Partner Type* (adaptive/ non-adaptive), two-way interactions *Partner* ^∗^ *Prime Structure* and *Partner Type* ^∗^ *Prime Structure*, and three-way interaction *Partner* ^∗^ *Partner Type* ^∗^ *Prime Structure*. The factor *Prime Structure* was dummy-coded (all means compared to reference group: intransitive primes). For the other two categorical factors we used sum-contrasts. Random intercepts were included for participants and items, and random by-item slopes for *Partner* and *Partner Type* (this is the maximal random effects structure for which convergence was reached; Barr et al., 2013).

## Results

There was a main effect of *Passive Prime Structures* on the production of passive targets (*p* < 0.001, Table 1): across all participant groups, participants used more passive sentences to describe target photographs after they had heard a passive prime sentence, relative to the baseline (intransitive prime). In line with the inverse preference effect reported frequently in the literature, there was no syntactic priming effect for actives.

**Table 1:** Results general linear mixed effects model.

	Coefficient	SE	Wald *Z*	*p*
Intercept	-1.85	0.11	-17.48	<0.001***
Active Prime	-0.09	0.06	-1.36	0.175
Passive Prime	0.56	0.06	9.34	<0.001***
Partner (Interlocutor / No interlocutor)	-0.14	0.09	-1.61	0.108
Partner Type (Adaptive / Non-adaptive)	-0.08	0.08	-0.92	0.359
Active Prime × Partner	0.06	0.07	-0.86	0.390
Passive Prime × Partner	0.13	0.06	2.22	0.026*
Active Prime × Partner Type	-0.05	0.07	-0.73	0.461
Passive Prime × Partner Type	0.01	0.06	-0.15	0.883
Partner × Partner Type	0.06	0.08	0.70	0.482
Active Prime × Partner × Partner Type	-0.06	0.06	-0.92	0.360
Passive Prime × Partner × Partner Type	0.00	0.06	0.02	0.986

^∗^*p < 0.05, ^∗∗^*p* < 0.01, and ^∗∗∗^*p* < 0.001*.

**Figure 2:**
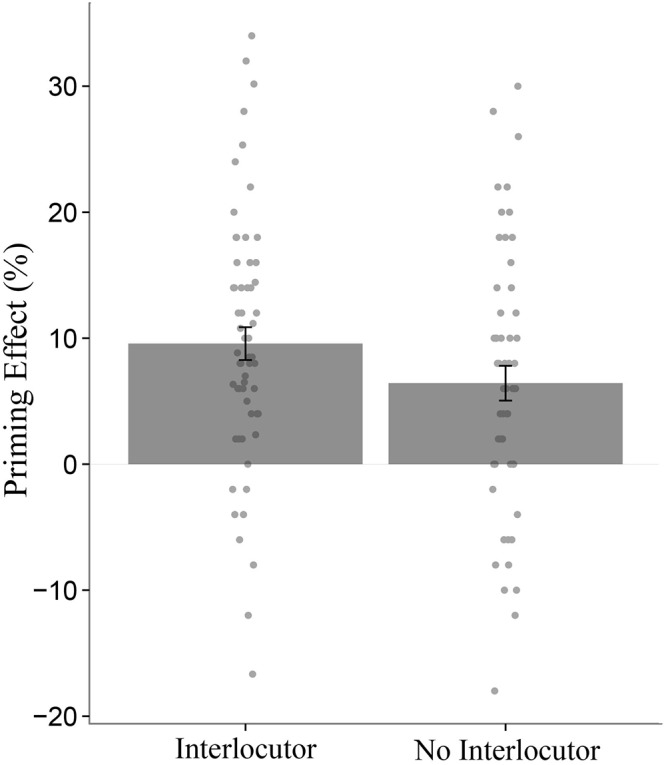
There was a stronger priming effect (% passive targets after a passive prime minus % passive targets after a baseline prime) in the Interlocutor conditions (left) compared to the No interlocutor conditions (right). Each dot represents one participant. Error bars represent standard error of the mean (SE).

**Figure 3:**
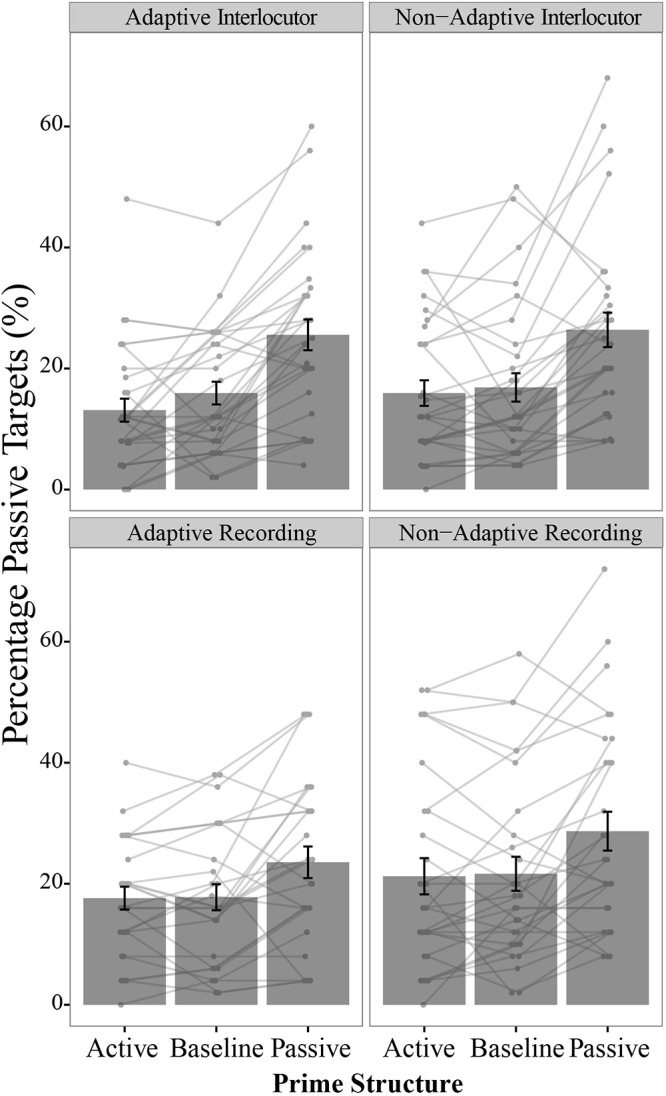
Percentage passive targets per participant group, per prime structure. Bars represent group mean per prime structure, error bars represent standard error of the mean (SE). Each dot represents one participant; connected dots are data points from the same participant. There was an effect of passives primes on syntactic choices overall, which was stronger for the Interlocutor compared to the No Interlocutor condition. We found no evidence for a difference between Adaptive and Non-adaptive conditions.

Although the effect of syntactic priming was present across all groups, it was stronger for participants in the Interlocutor conditions than for participants in the No Interlocutor conditions, as evidenced by a significant *Partner* ^∗^ *Prime Structure* interaction (*p* < 0.026, Table 1). This interaction is visualized in Figure 2. We found no evidence in line with the hypothesis that interacting with an adaptive partner increases a speaker’s own priming magnitude (relative to a non-adaptive partner): interactions *Partner Type* ^∗^ *Prime Structure* or *Partner* ^∗^ *Partner Type* ^∗^ *Prime Structure* were not significant (visualized in Figure 3).

## Discussion

In the present study, we measured the effect of syntactic priming on participants’ syntactic choices and compared the magnitude of the priming effect in a condition with an interlocutor versus condition with no interlocutor. Moreover, half of the participants in the interlocutor condition and in the no-interlocutor condition were paired with a “partner” who repeated their syntactic choices back to them (i.e., adaptive interlocutor or adaptive recording) and the other half was presented with a partner who was not “primed” by the participant (i.e., non-adaptive interlocutor or non-adaptive recording). We observed that (1) there was a syntactic priming effect for passives across all conditions, in line with the inverse preference effect; (2) syntactic alignment is stronger in the presence of an interlocutor than when no interlocutor is present (i.e., primed by a recording); (3) there was no evidence that a speaker’s syntactic priming magnitude is influenced by their conversation partner’s priming magnitude.

### Syntactic Priming and the Inverse Preference Effect

We replicated previous studies that have reported syntactic priming effects for passive/active alternations (Bock, 1986; Hartsuiker and Kolk, 1998; Bock and Griffin, 2000; Segaert et al., 2011). As expected based on this literature, we found significant syntactic priming effects for passives, but not actives. That is, participants produce significantly more passive sentence descriptions for target pictures following a passive prime sentence than for target pictures following a baseline prime, whereas they did not produce more active sentences after an active prime than after a baseline prime. In other words, there is an inverse preference effect: priming effects on syntactic choices are stronger for the less preferred alternative (Bock, 1986; Bock and Loebell, 1990; Hartsuiker and Kolk, 1998; Bernolet et al., 2009; Segaert et al., 2011, 2016). Implicit learning accounts of syntactic priming posit that less frequent syntactic structures are less expected and therefore accompanied by more prediction error, and greater changes in implicit knowledge, compared to more frequent syntactic structures (Chang et al., 2000, 2006, 2015; Jaeger and Snider, 2013).

### Syntactic Alignment Increases in the Presence of an Interlocutor

We found stronger syntactic priming effects in the interlocutor versus no interlocutor conditions. Participants in these two contexts performed exactly the same task: they described photographs and listened to descriptions of photographs. Across conditions, the number and distribution of primes and targets was identical. If syntactic priming is a purely low level, automatic effect of priming particular aspects in a linguistic utterance (here: sentence structure) on subsequent language production, we should not have observed any differences between these two groups. But we did find a difference: participants in the interlocutor conditions aligned more with their partner than participants in no interlocutor conditions did. This suggests that not only linguistic features but also top-down factors, such as having a communicative intent, affect the degree to which syntactic priming occurs.

To the best of our knowledge, this study is the first to compare syntactic alignment in the presence versus absence of an interlocutor. Other studies have compared syntactic priming magnitude when speakers were primed by a human or a computer, but crucially, in both cases, speakers were interacting with a partner. That is, both human and computer functioned as the participants’ addressee. In our study, the conditions differed on the degree to which a communicative goal was present for the speaker. In the Interlocutor Condition, the partner had to act based on the participant’s utterance (i.e., performance depends on communicative success: successful comprehension of what the speaker says). The presence of an interlocutor may elicit other top-down influences also (such as social goals), but we suggest our findings can at least in part be attributed to the speaker having a communicative goal. In the interlocutor conditions, participants may want to facilitate language processing for their partner. This would be in line with findings that alignment facilitates language comprehension (see also Branigan et al., 2010; Reitter et al., 2010; Jaeger and Snider, 2013).

However, there is one caveat to our explanation. By trying to make the difference between the interlocutor and no interlocutor conditions as strong as possible, we opted for a design in which the conversation partner in the interlocutor conditions was physically present. Therefore, the interlocutor and no interlocutor conditions did not merely differ in terms of having a communicative goal or not, but also in the physical presence/absence of a conversation partner. The presence of a conversation partner could have influenced syntactic alignment in ways which are not directly linked to communicative intent. We ensured that the confederate did not make co-speech gestures since these are known to show adaptation (Holler and Wilkin, 2011), which can facilitate language processing (Kelly et al., 2010). It is possible, however, that in the interlocutor conditions in our study, the participant and confederate aligned on lower levels of linguistic or non-linguistic behavior, and that alignment at these lower levels percolated up to alignment at the higher sentence level (suggested by Pickering and Garrod, 2004). If the confederate and participant aligned on lower levels of linguistic processing (e.g., intonation pattern, speech rhythm), this may have led to more alignment at higher levels, and thus more syntactic alignment. In contrast, recordings could not adapt to the participant on any levels. Future studies could isolate the influence of communicative intent on syntactic priming by comparing two groups of participants who perform a syntactic priming experiment in isolated, soundproof booths. In one group, participants would be led to believe that the recordings are actually live descriptions of another participant and that they are doing the task together. Crucially, participants should feel like they are actually communicating a message to their partner, so they should be provided with feedback about the partner’s response. If there is a difference between the magnitude of syntactic alignment in this group and a second group of participants who are told they are listening to recordings, we can be sure that this difference is due to having or not having an intention to communicate with a conversation partner.

Although a bottom-up contribution in the present study cannot be excluded, our findings suggest that very likely syntactic alignment cannot be fully explained by mechanisms that are encapsulated within the language system itself (Branigan et al., 2010). Accounts of syntactic alignment should therefore be able to incorporate top-down effects of being in a conversation context. We want to emphasize that we do not propose that facets of the communicative context would determine whether syntactic alignment occurs *per se*. Syntactic alignment is at least in part an automatic process that occurs due to facilitation in accessing representations, due to learning or a combination of both (Pickering and Branigan, 1998; Chang et al., 2006; Jaeger and Snider, 2013) and in line with this, in the present study, speakers show priming effects for passives in all conditions. However, the strength of the priming effect is mediated by the presence of an interlocutor.

### A Speaker’s Syntactic Priming Magnitude Is Not Influenced by Their Conversation Partner’s Priming Magnitude

We did not find evidence that the degree to which speakers align syntactic choices with their partner is affected by the syntactic priming magnitude of their partner (irrespective of whether that partner was a physically present person or a recording). Hence, contrary to our expectation, we did not find evidence that speakers who were paired with an adaptive partner (repetition of passive targets in 90% of the cases) were more strongly primed by that partner (more so than speakers who were paired with a non-adaptive partner - repetition in 10% of the cases). This finding could be interpreted in the context of studies demonstrating that there was no influence of participants’ syntactic structures being repeated by a confederate on participants’ prosocial behavior (Abrahams et al., 2018). It has been suggested that in contrast to lexical mimicry (Van Baaren et al., 2003), syntactic mimicry is not strong enough to induce prosocial behavior (Kulesza et al., 2013; Abrahams et al., 2018).

Although indeed it is possible that speakers are not influenced by the syntactic priming magnitude of their partner (contrary to what was suggested by Schoot et al., 2014 on reaction time syntactic priming effects in a conversation context), null results should always be interpreted with caution. One explanation for the fact that we did not find a difference between the two groups is that our critical manipulation depended on participants “spontaneously” producing passive descriptions of target photographs that were presented following baseline primes. Between subjects, we then manipulated whether the confederate would use a passive / a recording of a passive sentence was played (syntactic repetition). Although we added a training phase to the experimental procedure with the goal to increase the proportion of passives produced in the main experiment, and excluded participants who had not produced any passive targets after a baseline (and were thus not exposed to the manipulation at all), there was a lot of variation between participants with respect to how many passives they produced after a baseline prime. Consequently, there was a lot of variation in how much exposure participants had to the priming magnitude of their partner (the confederate or recording). On average, participants in the adaptive conditions only produced 8.75 passive targets (out of 50) following a baseline prime (minimum of 1 - maximum of 22, *SD* = 5.25). The manipulation of conversation partner’s (confederate or computer) degree of alignment was thus a very subtle manipulation in the present study.

## Conclusion

Our results suggest that there is a top-down influence of having an interlocutor, which increases syntactic alignment. Speakers’ syntactic priming effects are stronger when primes are provided by, and targets are addressed to an interlocutor than when primes are pre-recorded utterances and speakers produce targets without addressing someone. This suggests that syntactic priming cannot be fully explained by mechanisms that are encapsulated within the language system itself (Branigan et al., 2010) and calls for a mediated account of priming, in which the degree of priming can be modulated by top-down influences.

## Data Availability

The datasets generated for this study are available on request to the corresponding author.

## Author Contributions

LS, PH, and KS designed the study. LS analyzed the data. LS wrote the first draft of the manuscript. All authors revised the manuscript.

## Conflict of Interest Statement

The authors declare that the research was conducted in the absence of any commercial or financial relationships that could be construed as a potential conflict of interest.
